# Novel Bilayer-Shelled N, O-Doped Hollow Porous Carbon Microspheres as High Performance Anode for Potassium-Ion Hybrid Capacitors

**DOI:** 10.1007/s40820-023-01113-6

**Published:** 2023-06-07

**Authors:** Zhen Pan, Yong Qian, Yang Li, Xiaoning Xie, Ning Lin, Yitai Qian

**Affiliations:** 1 Department of Applied Chemistry, University of Science and Technology of China, Hefei, 230026 People’s Republic of China; 2 Yongjiang Laboratory, Ningbo, 315202 People’s Republic of China; 3 China National Building Material Design & Research Institute Co., Ltd., No. 208, Huazhong Road, Gongshu District, Hangzhou, 310022 People’s Republic of China

**Keywords:** Self-template method, Bilayer-shelled hollow porous structure, N, O-doped carbon microspheres, Dual-carbon potassium‐ion hybrid capacitor

## Abstract

With the advantages of high energy/power density, long cycling life and low cost, dual-carbon potassium ion hybrid capacitors (PIHCs) have great potential in the field of energy storage. Here, a novel bilayer-shelled N, O-doped hollow porous carbon microspheres (NOHPC) anode has been prepared by a self-template method, which is consisted of a dense thin shell and a hollow porous spherical core. Excitingly, the NOHPC anode possesses a high K-storage capacity of 325.9 mA h g^−1^ at 0.1 A g^−1^ and a capacity of 201.1 mAh g^−1^ at 5 A g^−1^ after 6000 cycles. In combination with ex situ characterizations and density functional theory calculations, the high reversible capacity has been demonstrated to be attributed to the co-doping of N/O heteroatoms and porous structure improved K^+^ adsorption and intercalation capabilities, and the stable long-cycling performance originating from the bilayer-shelled hollow porous carbon sphere structure. Meanwhile, the hollow porous activated carbon microspheres (HPAC) cathode with a high specific surface area (1472.65 m^2^ g^−1^) deriving from etching NOHPC with KOH, contributing to a high electrochemical adsorption capacity of 71.2 mAh g^−1^ at 1 A g^−1^. Notably, the NOHPC//HPAC PIHC delivers a high energy density of 90.1 Wh kg^−1^ at a power density of 939.6 W kg^−1^ after 6000 consecutive charge–discharge cycles.

**Supplementary Information:**

The online version contains supplementary material available at 10.1007/s40820-023-01113-6.

## Highlights

- Proposing a one-step pyrolysis strategy to fabricate a novel bilayer-shelled N, O-doped hollow porous carbon microspheres (NOHPC) anode.
- The optimized NOHPC anode displays a high K-storage capacity of 325.9 mAh g^−1^ at 0.1 A g^−1^ and excellent rate performance (201.1 mAh g^−1^ at 5 A g^−1^ after 6000 cycles).
- The assembled NOHPC//hollow porous activated carbon microspheres (HPAC) potassium ion hybrid capacitors deliver a high energy density of 90.1 Wh kg^−1^ at a power density of 939.6 W kg^−1^ even over 6000 cycles.

**Supplementary Information:**

The online version contains supplementary material available at 10.1007/s40820-023-01113-6.

## Graphical abstract

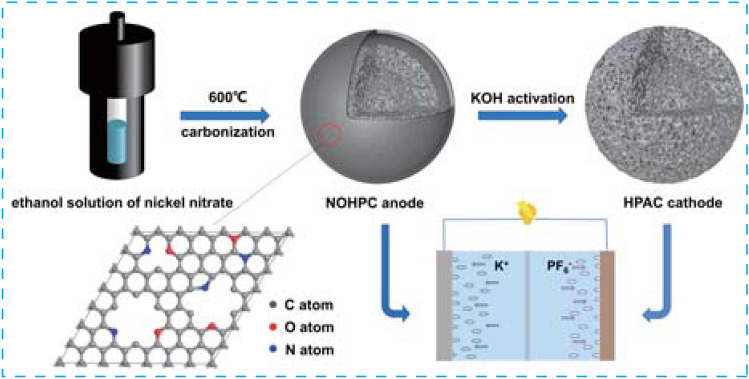

## Introduction

Benefited from the high energy/power density and low cost, dual-carbon potassium ion hybrid capacitors (DC-PIHCs) have been considered to be viable candidates for energy storage and conversion [1–5]. However, limited by the large radius of K^+^ (1.38 Å), the main challenge of DC-PIHCs is the rate performance and long-cycle performance of battery-type carbon anodes fail to match the dynamics and cycle stability of capacitive carbon cathodes [6–8]. For example, graphite anode provides a theoretical specific capacity of 279 mAh g^−1^ by forming the intercalation compound KC_8_, however, unsatisfactory rate performance and significant capacity degradation during cycling limit its further application [9, 10]. Thus, there is an urgent need for material innovation of carbonaceous anodes to ensure both higher reversible capacity and longer cycling life [11]. Meanwhile, it is necessary for DC-PIHCs to prepare capacitor‐type activated carbon cathodes with a high specific surface that provides high electrochemical adsorption capacity [12].

Recently, studies have shown that the heteroatom doping strategy significantly improves the electrochemical performance of carbon anodes by tuning their electronic structure and interlayer spacing [13–17]. In addition, constructing porous structure enables carbon anodes accommodate huge volume strain and exposes a large number of active sites for K^+^ storage [18–21]. For example, Wang et al. have obtained P, O-doped porous carbon spheres using MnO as template by a three-stage synthesis method, which exhibited excellent rate performance as PIHC anode materials [22]. In PIBs, Chong et al. synthesized a N, O-doped yolk-shell carbon sphere anode via a mixture strategy of soft-template and hydrothermal processes, which shows long cycling life with a capacity retention of 85.8% at 500 mA g^−1^ after 2500 cycles [23]. Lu et al. reported a facile precipitation polymerization and high-temperature carbonization approach for synthesis of a N-doped ball-in-ball structured hierarchical carbon microspheres as high performance anode for Na^+^ storage [24]. As mentioned above, the preparation of heteroatom-doped core-shell porous carbon anode underwent a series of complex synthesis processes. Therefore, it is necessary to design a novel three-dimensional carbon structure with heteroatom doping and large porosity through a simple synthesis process to further promote the application of DC-PIHC [25–28].

Herein, inspired by the solvothermal preparation of carbon materials, we proposed a novel self-template method for the preparation of bilayer-shelled N, O-doped hollow porous carbon microspheres (NOHPC) anode consisting of dense thin shell and hollow porous spherical core through one-step carbonization reaction [29]. Due to the template effect of NiO and the high-temperature catalysis of Ni^2+^, the NOHPC anode with bilayer-shelled structure has been synthesized by reaction of ethanol solution with nickel nitrate in stainless steel autoclave at 600 °C, which possesses a high capacity of 325.9 mAh g^−1^ at 0.1 A g^−1^ and a capacity of 201.1 mAh g^−1^ at 5 A g^−1^ over 6000 cycles. In combination with ex situ Raman, galvanostatic intermittent titration technique (GITT), and density functional theory (DFT) calculations, the high reversible capacity has been demonstrated to be attributed to the co-doping of N/O heteroatoms and porous structure improved K^+^ adsorption and intercalation capabilities, and the stable long-cycling performance originating from the bilayer-shelled hollow porous carbon sphere structure. In addition, the high specific surface area hollow porous activated carbon spheres (HPAC) were converted by KOH activation of NOHPC, which has excellent rate performance and high specific capacity (71.0 mAh g^−1^ at 1 A g^−1^ after 5000 cycles) as PIHCs cathode. The assembled NOHPC//HPAC PIHC delivers a high energy density of 90.1 Wh kg^−1^ with only 0.007% capacity decay per cycle at a power density of 939.6 W kg^−1^ even over 6000 cycles.

## Experimental Section

### Material Synthesis

#### Synthesis of NOHPC, NOCB and NOCNT

Firstly, 2.9 g nickel nitrate hexahydrate is dissolved in 10 mL anhydrous ethanol to prepare about 1 M nickel nitrate ethanol solution. Then, NOHPC was obtained by high-temperature ethanol thermal reduction reaction with 10 mL nickel nitrate ethanol solution in 20 mL stainless steel autoclave. The autoclave was heated at 700 °C with a heating rate of 5 °C min^−1^, and the reaction pressure is about 100 MPa. After 5 h, the samples were taken out from autoclave which was naturally cooled to room temperature. Washed with hydrochloric acid and deionized water, the NOHPC powder was dried under vacuum at 60 °C for 12 h. Use the same synthesis method, when the carbonization temperatures were 600 and 800 °C, NOCB and NOCNT were obtained.

#### Synthesis of HPAC

As-prepared NOHPC and activation agent (KOH) were thoroughly mixed with the mass ratio of 1:3. The mixtures were put in a tubular furnace and heated at 800 °C for 2 h under argon atmosphere with a heating rate of 5 °C min^−1^. The activated product was washed with hydrochloric acid to remove extra activation agent, and then washed with deionized water for several times. Finally, the dark product was dried in an oven at 60 °C overnight.

### Material Characterization

The structures of the samples were measured by X-ray diffraction (XRD) on a Philips X’ Pert Super diffractometer with Cu K_α_ (*λ* = 1.54182 Å), and Raman spectroscopy was performed by a JYLABRAM-HR Confocal Laser Micro-Raman spectrometer at 532 nm. The morphologies of the samples were characterized on scanning electron microscopy (SEM, JEOL-JSM-6700F), transmission electron microscopy (TEM, Hitachi H7650) and high-resolution transmission electron microscopy (HRTEM, JEM-2100F). The surface areas and pore size distribution of the samples were obtained by BEL SORP-max machine (BEL, Japan). Thermogravimetric analysis (TGA) was carried out on Shimadzu TGA-50H. The FTIR spectra is tested on Fourier transformed infrared spectrometer (Hyperion 3000). X-ray photoelectron spectroscopy (XPS) was collected on an ESCALAB 250 X-ray photoelectron spectrometer (PerkinElmer).

### Electrochemical Measurements

The electrochemical performances of the samples were measured using CR2016 coin cells with about 150 μL electrolyte (0.8 M KPF_6_ in ethylene carbonate (EC) and propylene carbonate (PC) (1:1, v/v)). The anode was composed of 70 wt% active materials, 20 wt% super P, 10 wt% carboxymethyl cellulose (CMC). The mixed slurry was coated on a copper film and dried at 100 °C for 5 h in a vacuum oven. The average active material loading of anodes was calculated at about 1.0 mg cm^−2^. And the coin cells were assembled in the argon-filled glove box (O_2_, H_2_O < 0.1 ppm). The discharge and charge measurements were carried out at various current rates in the voltage range of 0.01–2.5 V on a LANDCT2001A battery tester. And the cathode was prepared by mixing active materials into super P and PVDF with a weight ratio of 8:1:1 in NMP, followed by pasting the slurry onto an aluminum foil. The average active material loading of cathodes was calculated at about 2.5 mg cm^−2^. The cathodes were cycled at 1.2–4.0 V, while PIHCs were cycled at 0.5–3.8 V. Half cells of the PIBs were assembled in glovebox under Ar with active materials as the anode, potassium foil as the counter electrode, and the PIHC were assembled with NOHPC as the anode, HPAC as the cathode. The cyclic voltammogram (CV) measurements were performed on a CHI 660D Electrochemical Workstation (Shanghai Chenhua Corp.).

In PIHCs full-cell tests, the calculations of energy (*E*, Wh kg^−1^) and power densities (*P*, W kg^−1^) based on the total mass of both anode and cathode materials are performed using Eq. 1:

1 $$E = P \times t/3600$$

in which* t*(s) is the discharge time, *E*(Wh kg^−1^) is the specific energy in the discharge phase from the LANDCT2001A battery tester.

## Results and Discussion

### Formation Mechanism and Structural Characterization

**Fig. 1:**
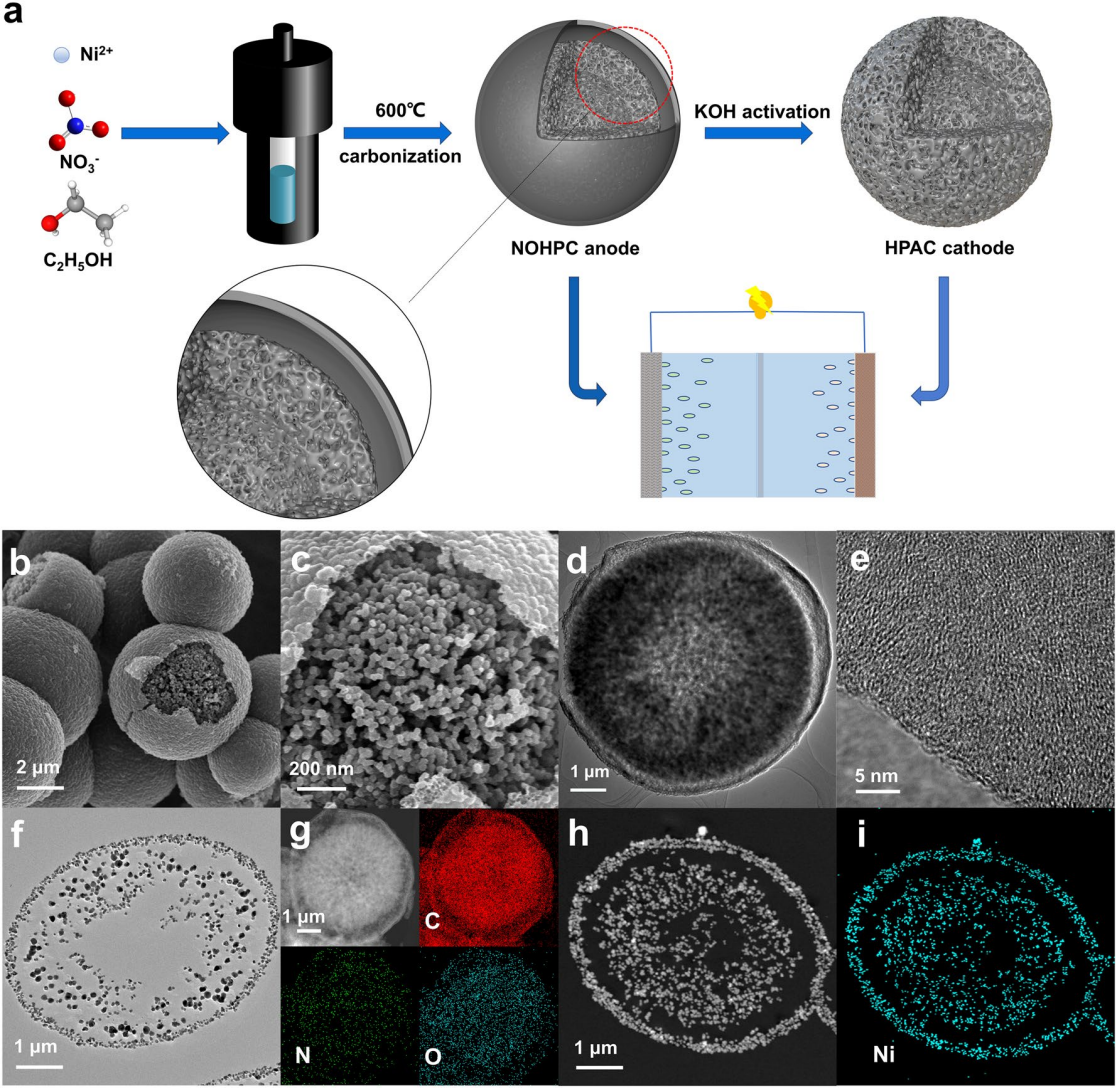
**a** Schematic illustrations of the formation and microstructure of NOHPC and HPAC. **b**, **c** SEM images of NOHPC. **d** TEM image of NOHPC. **e** HRTEM image of NOHPC. **f** TEM images of ultrathin section for NOHPC. **g** EDS elemental mapping images of NOHPC (Red, green and blue represent the C, O, and N, respectively). **h**, **i** EDS elemental mapping images of ultrathin section for unwashed NOHPC

Schematic illustration of the synthetic process and the morphological evolution of the novel bilayer-shelled N, O-doped hollow porous carbon microspheres (NOHPC) and the hollow porous activated carbon spheres (HPAC) are shown in the Fig. 1a. In a typical carbonization process taken place in the stainless steel autoclave, the ethanol solution of nickel nitrate were converted into bilayer-shelled hollow porous carbon microsphere, meanwhile the Ni^2+^ were in-situ turned into NiO template. After being washed with hydrochloric acid and deionized water, the NOHPC consisting of a dense thin shell and a hollow porous spherical core was obtained (Figs. 1b–d and S1). Then, the HPAC with high specific surface area were prepared by KOH activation of NOHPC. Furthermore, the high-resolution transmission electronic microscope (HRTEM) images of NOHPC display that the inner hollow porous spherical core composed of small particles exhibits the low crystallinity, which is conducive to the rapid shuttle of potassium ions in carbon materials (Figs. 1e and S2). In order to visually observe the hollow and porous bilayer-shelled structure, we prepared NOHPC slices with a thickness of 70 nm by gradient resin infiltration and ultrathin sectioning technology. In Fig. 1f, TEM image of ultrathin section for NOHPC displays a novel bilayer-shelled carbon spheres composed of a dense shell and a loose hollow porous core, in which the core part contributes to high K-storage capacity, and the shell part ensures the structural stability of microspheres during charge and discharge process [30–33]. The energy dispersive spectrometer (EDS) mapping images confirm the uniform distribution of N, O elements in NOHPC, illustrating that the N, O element in the nitrate ion have been doped into the samples after the pyrolysis process (Fig. 1g). To explore the role of Ni^2+^ in the preparation of carbon materials, we prepared the unwashed NOHPC slices with a thickness of 70 nm by gradient resin injection and ultrathin sectioning technology. In Figs. 1h–i and S3, the EDS mapping images prove that Ni and O elements are uniformly distributed in unwashed NOHPC.

**Fig. 2:**
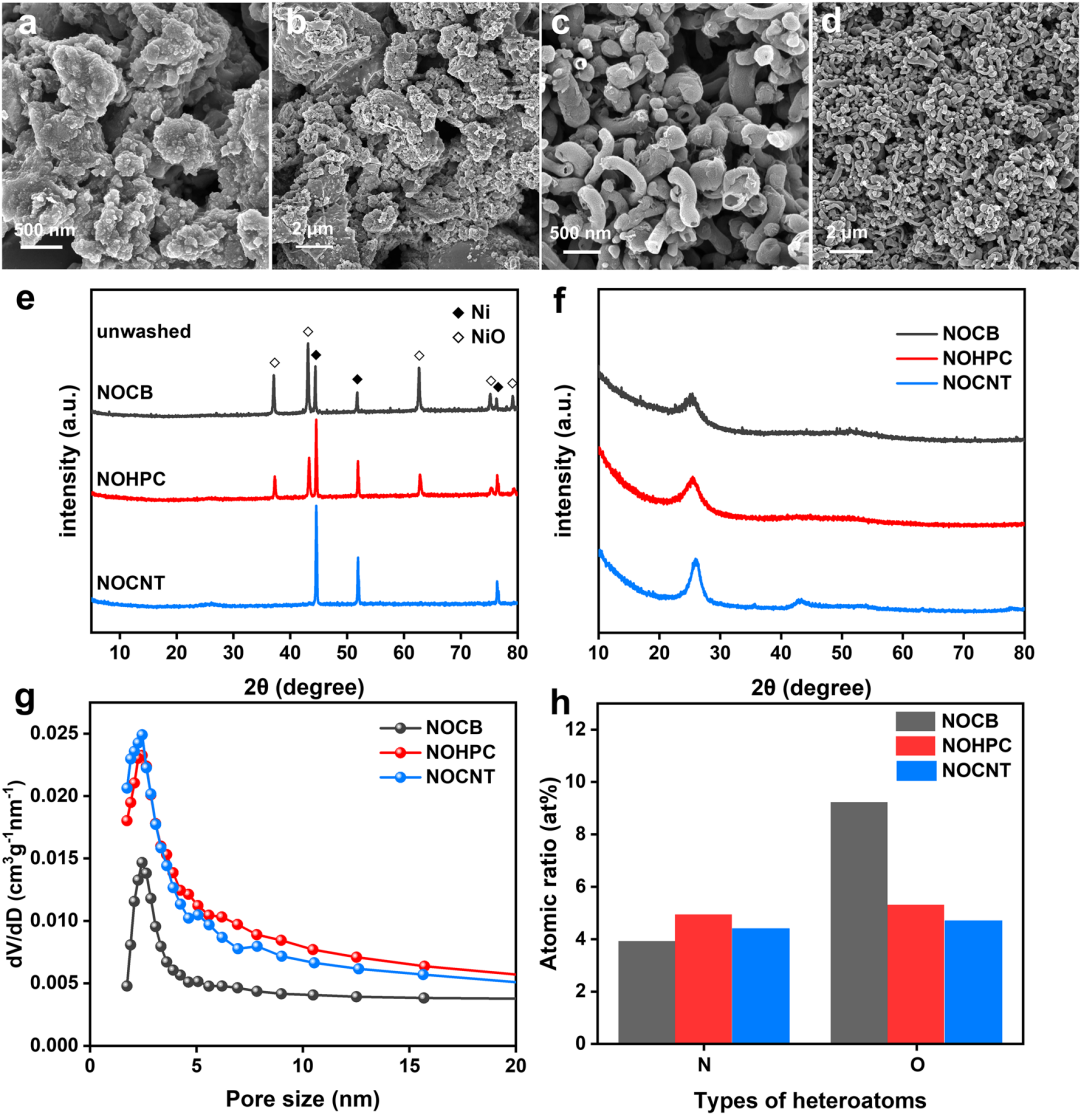
**a**, **b** SEM images of NOCB. **c**, **d** SEM images of NOCNT. XRD patterns of NOCB, NOHPC, and NOCNT **e** before and **f** after washing with 1 M hydrochloric acid solution and deionized water. **g** BJH pore width of NOCB, NOHPC, and NOCNT. **h** XPS results of the N, O atomic ratio of NOCB, NOHPC, and NOCNT

In order to explore the formation mechanism of NOHPC, a series of N, O-doped carbon materials were prepared in the same autoclave by adjusting the carbonization temperature. As shown in Fig. 2a–d, N, O-doped irregular carbon blocks (NOCB), and N, O-doped carbon nanotubes (NOCNT) were generated by pyrolysis of the ethanol solution of nickel nitrate in stainless steel autoclaves at 500 and 700 °C, respectively. Clearly, a group of diffraction peaks (labeled as ◇) in the pattern are characterized as NiO (JCPDS No. 73–1523), which weakens with increasing carbonization temperature and disappears at 700 °C (Fig. 2e, f). In contrast, the diffraction peaks of Ni (labeled as ◆, JCPDS No. 04–0850) are stronger with increasing carbonization temperature. Therefore, the transformation of Ni^2+^ into NiO forms hollow porous bilayer-shelled structures, and the further reduction in NiO results in the formation of uniform carbon nanotubes. In conclusion, Ni^2+^ not only generates NiO template, but also plays a catalytic role in the carbonization process. In addition, it can be found that NOCNT exhibits the highest degree of graphitization with the highest intensity of diffraction peaks near 25° and 43° corresponding to the crystallographic planes of (002) and (100) compared to samples obtained at other temperatures. The Brunauer–Emmett–Teller (BET) isotherms of NOCB, NOHPC, and NOCNT were assessed by nitrogen adsorption/desorption isotherms. As shown in Fig. S4, the BET surface areas of NOCB, NOHPC, and NOCNT are 99.95, 185.56, and 186.68 m^2^ g^−1^, respectively. And the Barrett–Joyner–Halenda (BJH) pore widths of the carbon materials are concentrated in the < 5 nm range, which ensures rapid K^+^ diffusion during the charge/discharge process (Fig. 2g). Raman spectra shows two characteristic peaks at 1360 cm^−1^ (D band) and 1585 cm^−1^ (G band), which correspond to the vibration of disordered carbon and the in-plane C–C bond stretching vibration of crystalline graphite, respectively [34]. In Fig. S5, the Raman details spectra and fitting results show the *I*_G_/*I*_D_ of NOCB, NOHPC, and NOCNT are 0.436, 0.443, and 0.503, respectively, denoting the existence of significant structural defects in the samples [35]. Moreover, the X-ray photoelectron spectroscopy (XPS) survey scans demonstrate the presence of C, N, and O in NOCB, NOHPC, and NOCNT (Fig. S6). The oxygen atomic ratio of NOCB, NOHPC and NOCNT are 9.21, 5.28, and 4.70 at%, and the nitrogen atomic ratio of NOCB, NOHPC, and NOCNT are 3.89, 4.91, and 4.40 at%, respectively (Fig. 2h). As shown in Fig. S7, the deconvolution N 1* s* spectrum includes three peaks at 398.7, 400.1, and 401.4 eV, representing pyridinic nitrogen (N-6), pyrrolic N (N-5), and quaternary nitrogen (N-Q), respectively [36]. The presence of N-5, N-6 and O generates active sites and promotes the K^+^ intercalation process, resulting in improving the electrochemical properties of the carbon anode, and N-Q enhances the electronic conductivity [37–39].

### Electrochemical Performance and Structural Evolution

**Fig. 3:**
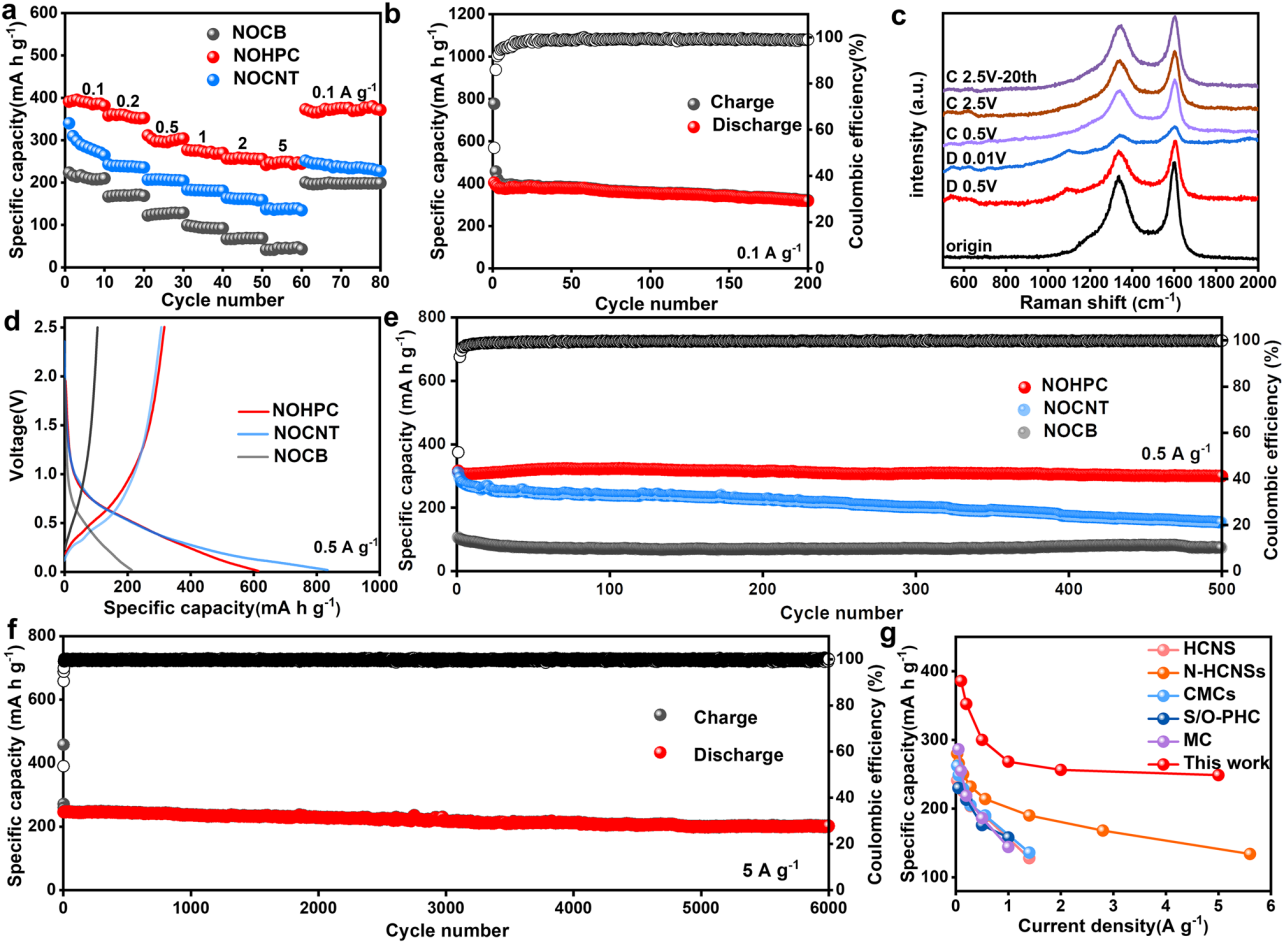
The electrochemical evaluation of NOCB, NOHPC and NOCNT anodes for PIBs, and the metallic potassium foil was used as both the counter and reference electrodes. **a** Rate capabilities at various current rates of NOCB, NOHPC and NOCNT. **b** Cycling performance of NOHPC at 0.1 A g^−1^. **c** Ex situ Raman spectra in the first discharge/charge process of NOHPC at 0.1 A g^−1^. **d** The first charge–discharge curves of NOCB, NOHPC and NOCNT at 0.5 A g^−1^. **e** Cycling performance of NOCB, NOHPC and NOCNT at 0.5 A g^−1^. **f** Cycling performance of NOHPC at a high current rate of 5 A g^−1^. **g** Electrochemical performances of the NOHPC and previously reported carbonaceous materials

To evaluate the electrochemical performance of bilayer-shelled NOHPC anode, a range of charge–discharge tests have been conducted in 0.8 M KPF_6_ in ethylene carbonate (EC) and propylene carbonate (PC) (v/v = 1:1) with potassium metal as a counter electrode during the voltage range of 0.01–2.5 V. Rate performances have been assessed at the current rates from 0.1 to 5 A g^−1^, and the reversible capacities of NOHPC are 385.9, 352.4, 300.1, 268.3, 256.4, and 248.8 mAh g^−1^ at the current densities of 0.1, 0.2, 0.5, 1, 2, and 5 A g^−1^, respectively (Fig. 3a). In contrast, the NOCB and NOCNT electrodes supply capacities of 45.8 and 138.8 mAh g^−1^ at 5 A g^−1^, proving that NOHPC proffers the best rate performance. As shown in Fig. 3b, the reversible capacity of the NOHPC is 405.5 mAh g^−1^ at a current density of 0.1 A g^−1^ with an ICE of 52.2% at the first cycle and a reversible capacity of 321.7 mAh g^−1^ is reserved over 200 cycles. Figure 3c shows ex situ Raman spectra of NOHPC anode at 0.1 A g^−1^. It is explicit that the D and G bands of NOHPC are both weakened at fully discharge state because of the intercalation of K^+^ [40–42]. And the D bands and G bands reappear during the depotassiation process, demonstrating excellent structural stability. Surprisingly, the D and G bands remain almost unchanged after 20 cycles at 0.1 A g^−1^, which corresponds to the remarkable long cycling stability of NOHPC.

In addition, at a current density of 0.5 A g^−1^, the NOCB, NOHPC, and NOCNT deliver the reversible capacities of 287.4, 316.7, and 308.6 mAh g^−1^ with ICEs of 48.7%, 51.5%, and 36.9% during the first charge and discharge cycle (Fig. 3d). After 500 cycles, NOHPC retains a specific capacity of 299.8 mAh g^−1^ with a loss of 5.3% compared with the first cycle, while NOCB and NOCNT only maintain capacities of 53.7 and 151.6 mAh g^−1^ (Fig. 3e). Notably, after 500 cycles at the constant current density of 0.5 A g^−1^, the morphology of the NOHPC was not significantly damaged, which demonstrates that the special hollow porous bilayer-shelled structure not only provides high electrochemical K-storage capacity but also maintains structural stability during cycling (Fig. S8). Exhilaratingly, at a high current rate of 5 A g^−1^, NOHPC offers a reversible capacity of 246.0 mAh g^−1^ in the first cycle and retains a reversible capacity of 202.6 mAh g^−1^ with a loss of 17.6% even over 6000 cycles (Fig. 3f). As shown in Fig. 3g and Table S1, a comparison with the electrochemical performance of other previously reported carbon anodes for PIBs indicates that the NOHPC anode has higher reversible capacity, excellent rate performance, and long cycling life [43–47].

### Kinetics Analysis and DFT

**Fig. 4:**
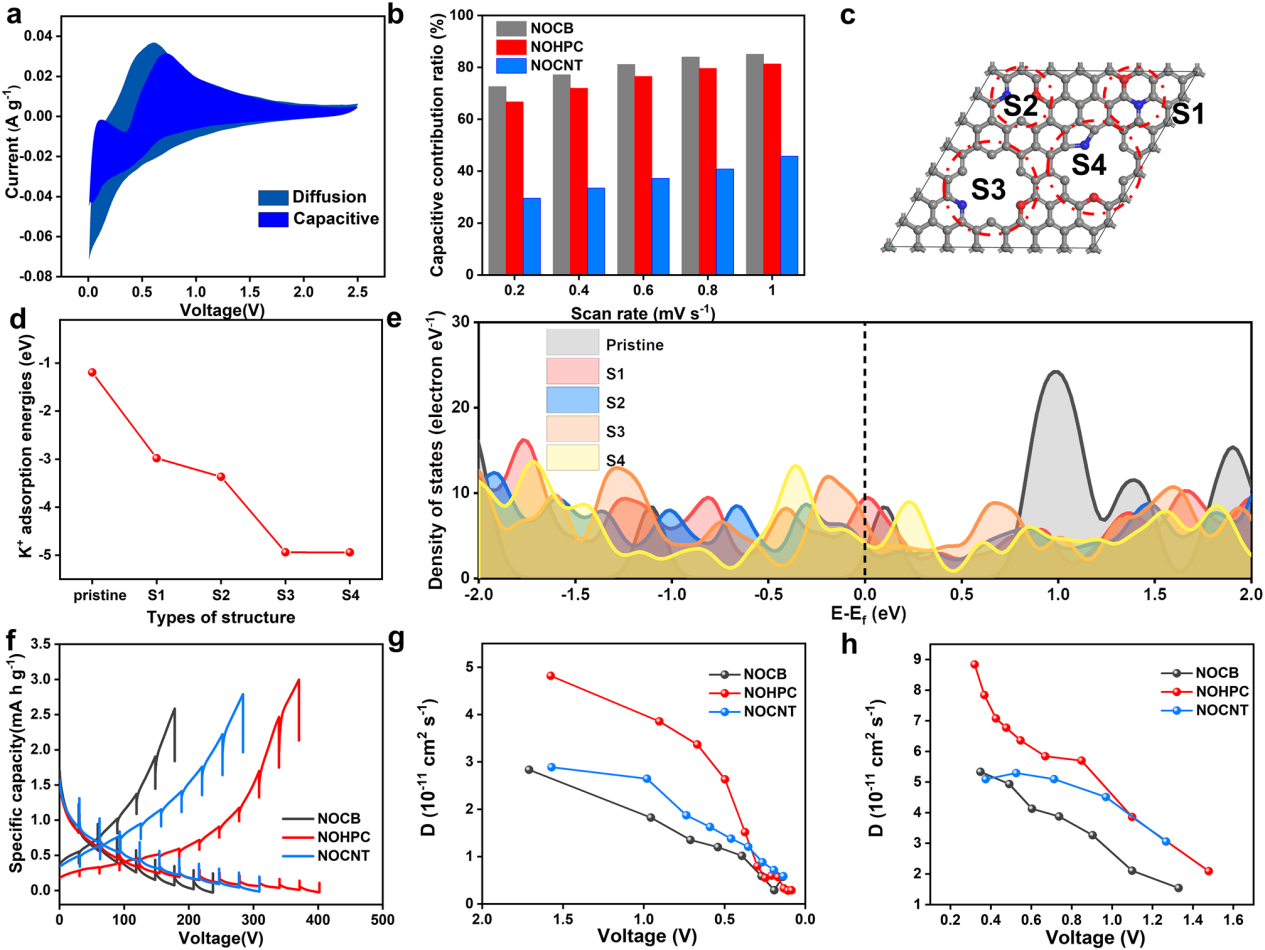
**a** Capacitive-contribution of NOHPC at the scan rate of 0.4 mV s^−1^. **b** Contribution ratios of adsorption capacity at different scan rates of NOCB, NOHPC, and NOCNT. Theoretical simulations and relative verifications: **c** Computational models of NOHPC (four types of K^+^ adsorption site labeled as S1, S2, S3, and S4, black, red, and blue represent the C, O, and N, respectively); **d** K^+^ adsorption energies of the pristine and four types of N, O-doped carbon structures; **e** DOS of the pristine and four types of N, O-doped carbon structures. **f** GITT curves of NOCB, NOHPC, and NOCNT at the second cycle. **g** Diffusion coefficients of the discharging process are calculated from the GITT curves as a function of cell voltage. **h** Diffusion coefficients of the charging process are calculated from the GITT curves as a function of cell voltage

The kinetic processes of NOCB, NOHPC, and NOCNT have been analyzed to reveal the reasons for outstanding electrochemical performance of NOHPC. The scanning CV curves of NOCB, NOHPC, and NOCNT at different scanning rates ranging from 0.2 to 1.0 mV s^−1^ are shown in Fig. S9, the similar curve shape illustrates the homologous K^+^ storage mechanism for anodes. The values of *b* (*i* = *av*^*b*^, *b* = 1 for the ideal capacitive behavior and *b* = 0.5 for the diffusion-limited process) for NOCB, NOHPC, and NOCNT are 0.910, 0.857, and 0.730, respectively, which indicates that the ion diffusion rate of carbon materials decreases with the increase in carbonization temperature at the voltage of peak current (Fig. S10). The capacitive contribution was calculated based on *i* = *k*_1_*v* + *k*_2_*v*^1/2^, where *k*_1_*v* is the capacitive contribution and *k*_2_*v*^1/2^ represents the diffusion-limited contribution [48, 49]. The capacitive contribution rate of 71.95% confirms that the charge storage of NOHPC dominated by the surface-driven capacitive behavior, which is instrumental to the high-rate performance and long-cycle stability (Fig. 4a). Notably, the capacitance contribution of NOCB and NOHPC exceed 60% at different scan rates, which indicates that the capacitance contribution to capacity is higher than the diffusion-limited contribution (Fig. 4b). On the contrary, for the NOCNT anode, the capacitance contribution to capacity is lower than the diffusion-limited contribution.

In order to further explain the excellent K^+^ storage performance of NOHPC, the K^+^ adsorption energy (Δ*E*_a_) and density of states (DOS) distributions for different N, O-doped structures have been obtained from first-principles calculations based on the density functional theory (DFT) [23, 50]. According to experimental data, a 8 × 8 × 1 supercell was constructed to simulate carbon material, while nitrogen and oxygen atoms were set into this 8 × 8 × 1 supercell to form the N, O-doped carbon material. In Fig. 4c, the carbon calculation model including pyrrolic nitrogen and oxygen-doped, pyridinic nitrogen and oxygen-doped, quaternary nitrogen and oxygen-doped carbon structures (donated as S1, S2, S3, and S4 represent the N-Q/O, N-6-1/O, N-6-2/O, and N-5/O, respectively) was constructed based on the XPS results of the current work, where the atomic ratio of C, N, and O are 90%, 5%, and 5%, respectively, while the N-5, N-6 and N-Q ratios are 1:2:1. In Figs. 4d and S11, the calculated K^+^ adsorption energies(ΔE_a_) for S1, S2, S3, and S4 are − 2.98, − 3.37, − 4.93, and − 4.94 eV, respectively, which are higher than the K^+^ adsorption energy of − 1.19 eV for pristine carbon. The results verify that the stronger K^+^ adsorption tendency induced by N/O doping contributes to the enhanced K^+^ storage capacity of the NOHPC anode. In addition, the DOSs of different carbon structures have been investigated (Fig. 4e). The increased DOS values of N/O-doped carbon structures (combined with K atom) around the Fermi energy level demonstrate higher electronic conductivity compared to undoped carbon layers (combined with K atom). The highest DOS value for the N-Q/O doping is attributed to the ability of graphitic-N to increase the electrical conductivity of the carbon material [51]. Thus, the N/O doping enhances K^+^ adsorption and electronic conductivity of carbon materials, resulting in excellent electrochemical performance of NOHPC as PIHCs anode.

In addition, it is important to explore the kinetic processes by galvanostatic intermittent titration technique (GITT). As shown in Fig. 4f, the GITT curve of NOHPC displays the lowest overpotential during the (de)potassiation period, which represents excellent kinetic performance [34]. During the potassiation process, the diffusion coefficient of NOHPC is significantly higher than that of NOCB and NOHCNT when the voltage is higher than 0.5 V (Fig. 4g). Meantime, the diffusion coefficient of NOHPC is higher than that of NOCB and NOCNT when the voltage is less than 1 V in the depotassiation process (Fig. 4h). In conclusion, the ion diffusion coefficient of NOHPC is generally higher than that of NOCB and NOCNT during the charge/discharge cycle, indicating bilayer-shelled hollow porous structure promote the transmission of ions.

### Electrochemical Performance of DC-PIHCs

With regard to DC-PIHCs, the capacitance of the carbon cathode is closely related to the specific surface area and pore structure [52–54]. Here, hollow porous activated carbon microspheres (HPAC) have been prepared as PIHC cathodes by KOH etching of NOHPC at 800 °C. As shown in Fig. S12, the spherical structure of NOHPC has been maintained after KOH activation, but the outer dense shell has been etched into a porous structure with abundant mesopores and micropores uniformly distributed on the carbon spheres. Meanwhile, the (002) peak in the XRD spectrum of HPAC disappears, indicating that KOH destroys the microcrystalline structure and creates abundant defects (Fig. S13). In Fig. S14, the nitrogen adsorption–desorption curves of HPAC is the typical type I isotherms, corresponding to a large number of micropores in the carbon material. Additionally, the BJH pore width of the sample is mainly concentrated in the range of < 4 nm in Fig. S15.

**Fig. 5:**
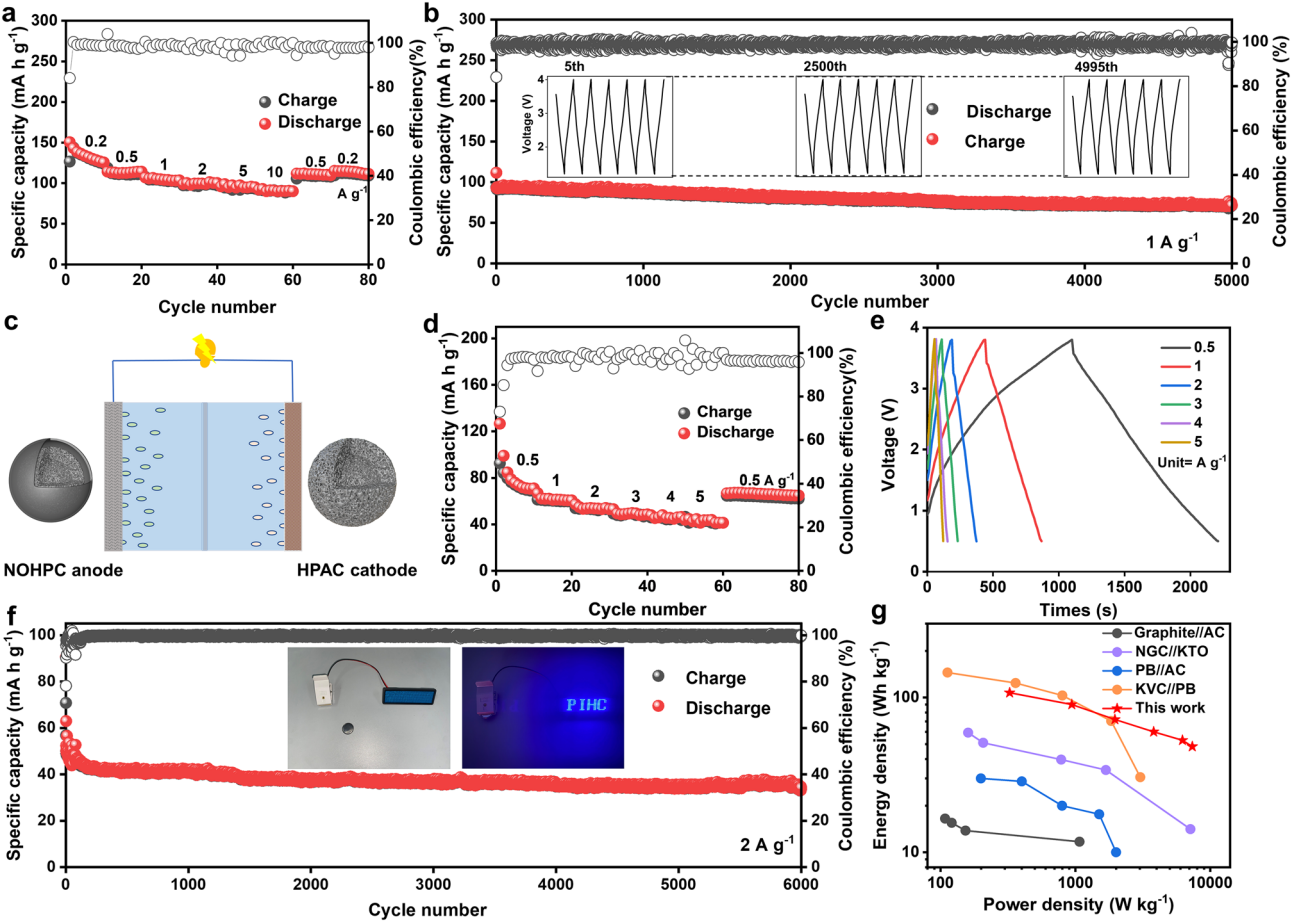
**a** Rate capabilities at various current rates of HPAC//K cell in the voltage range of 1.2–4.0 V. **b** Cycling performance of HPAC//K cell at 1 A g^−1^. **c** A schematic diagram of PIHC. **d** Rate capabilities at various current rates of NOHPC//HPAC PIHC in the voltage range of 0.5–3.8 V (based on the total mass of the active materials). **e** Charge/discharge curves at the different current densities of the NOHPC//HPAC PIHC (based on the total mass of the active materials). **f** Long-cycling performance of NOHPC//HPAC PIHC at a high current rate of 2 A g.^−1^ (based on the total mass of the active materials). **g** Ragone plots of the NOHPC//HPAC PIHC compared with previously reported PIHCs (based on the total mass of the active materials)

Then, the galvanostatic charge–discharge tests which used HPAC as the cathode have been carried out in the voltage range of 1.2–4.0 V. In Fig. 5a, the reversible capacities of HPAC cathode are 126.9, 113.1, 104.1, 99.6, 95.0, and 90.1 mAh g^−1^ at current densities of 0.2, 0.5, 1, 2, 5, and 10 A g^−1^, respectively. While returning to 0.5 and 0.2 A g^−1^, the reversible capacities of 111.0 and 114.9 mAh g^−1^ have been maintained after a range of high current rate cycles. Excitingly, at a constant current density of 1 A g^−1^, the HPAC cathode has a reversible capacity of 71. 2 mAh g^−1^ after 5000 consecutive charge–discharge cycles (Fig. 5b).

Furthermore, to appraise the feasibility of NOHPC anode and HPAC cathode for PIHC, a dual‐carbon NOHPC//HPAC PIHC device has been constructed (Fig. 5c). In Fig. 5d, at the current densities of 0.5, 1, 2, 3, 4, and 5 A g^−1^, the reversible capacities of PIHC are 67.7, 55.3, 47.7, 43.4, 38.9, and 35.4 mAh g^−1^ in the voltage range of 0.5–3.8 V. The GCD curves are triangular at different current densities, which indicates that Faraday processes have occurred during the charging-discharging of PIHC (Fig. 5e). As shown in Fig. 5f, the PIHC offers a reversible capacity of 40.9 mAh g^−1^ and retains a reversible capacity of 35.7 mAh g^−1^ with high Coulombic efficiency at 2 A g^−1^ after 6000 cycles. It is notable that a viable PIHC successfully lights up a “PIHC” panel consisting of light-emitting diodes (LEDs) after fully charging, as shown in the inset of Fig. 5f. In Fig. 5g, the comprehensive indexes of energy density and power density of NOHPC//HPAC PIHC (90.1 Wh kg^−1^ at 939.6 W kg^−1^, 52.93 Wh kg^−1^ at 6,217.5 W kg^−1^) surpass most of the previously reported PIHCs, such as AC//Graphite, NGC//KTO, PB//AC, KVC//PB [54–57].

## Conclusion

Herein, a novel self-template method has been developed for the preparation of high-performance bilayer-shelled structure NOHPC anode through one-step carbonization reaction. Then, HPAC cathode with high specific surface area (1,472.65 m^2^ g^−1^) has been converted by KOH activation of NOHPC. As a result, the optimized NOHPC anode with high N, O doping level (4.91 at% of N, 5.28 at% of O) displays a high reversible capacity of 299.8 mAh g^−1^ at 0.5 A g^−1^ after 500 cycles, outstanding rate capability, and a long cycle life of 6000 cycles with a high capacity of 202.6 mAh g^−1^ at 5 A g^−1^. In combination with ex situ Raman, GITT and DFT, the high reversible capacity has been demonstrated to be attributed to the co-doping of N/O heteroatoms and porous structure improved K^+^ adsorption and intercalation capabilities, and the stable long-cycling performance originating from the bilayer-shelled hollow porous carbon sphere structure. Meanwhile, the HPAC cathode with ultrahigh specific surface obtains superior rate performance and high reversible capacity (71.2 mAh g^−1^ at 1 A g^−1^). Finally, the assembled NOHPC//HPAC PIHC provides a reversible capacity of 40.9 mAh g^−1^ and maintains a reversible capacity of 35.7 mAh g^−1^ after 6000 cycles at 2 A g^−1^. It is notable that a viable PIHC successfully lights up a “PIHC” panel consisting of LEDs after fully charging, which signifies that the method offers a viable idea for the development of high performance PIHC.

### Supplementary Information

Below is the link to the electronic supplementary material.

Supplementary file1 (PDF 1317 kb)
