# Supplementary material for: Novel Bilayer-Shelled N, O-Doped Hollow Porous Carbon Microspheres as High Performance Anode for Potassium-Ion Hybrid Capacitors
Source: Nanomicro Lett. 2023 Jun 7;15:151. doi: 10.1007/s40820-023-01113-6 (PMC10247926; doi:10.1007/s40820-023-01113-6)
Supplement: Supplementary file 1 — Supplementary file1 (PDF 1317 kb) [file 40820_2023_1113_MOESM1_ESM.pdf]

Supporting Information for

Novel Bilayer-Shelled N, O-Doped Hollow Porous Carbon Microspheres as High Performance Anode for Potassium-Ion Hybrid Capacitors

Zhen Pan[1], Yong Qian[1], Yang Li[1], Xiaoning Xie[3], Ning Lin[1, 2, *], Yitai Qian[1]

[1]Department of Applied Chemistry University of Science and Technology of China Hefei 230026, P. R. China

[2]Yongjiang laboratory, Ningbo 315202, P. R. China

[3]China National Building Material Design & Research Institute Co., Ltd. No.208, Huazhong Road, Gongshu District, Hangzhou 310022, P. R. China

*Corresponding author. E-mail: ningl@mail.ustc.edu.cn (Ning Lin)

S1 Chemicals

Anhydrous ethanol and hydrochloric acid were purchased from Sinopharm Chemical Reagent Co., Ltd. Nickel nitrate hexahydrate was obtained from Aladdin. All chemicals were analytical grade and used as received. All aqueous solutions were prepared with ultrapure water from a Water Purifier System.

S2 DFT Calculations

All the calculations are performed in the framework of the density functional theory with the projector augmented plane-wave method, as implemented in the Vienna ab initio simulation package [S1]. The generalzied gradient approximation proposed by Perdew, Burke, and Ernzerhof is selected for the exchange-correlation potential [S2]. The long range van der Waals interaction is described by the DFT-D3 approach [S3]. The cut-off energy for plane wave is set to 400 eV. The energy criterion is set to 10^{-5} eV in iterative solution of the Kohn-Sham equation. A vacuum layer of 15 Å is added perpendicular to the sheet to avoid artificial interaction between periodic images. The Brillouin zone integration is performed using a 2×2×1 k-mesh. All the structures are relaxed until the residual forces on the atoms have declined to less than 0.02 eV $Å^{-1}$.

Supplementary Figures and Tables

Table S1 Comparison of electrochemical performance of the NOHPC anode with previous reports

Sample	Voltage range	Current	Capacity	References
hollow carbon nanospheres	0.01-2.0V	28 mA g^{-1}	100 cycles, 241.2 mA h g^{-1}	[S4]
N-doped hierarchical porous hollow carbon spheres	0.01-2.5V	1400 mA g^{-1}	600 cycles, ~140.0 mA h g^{-1}	[S5]
hard carbon microspheres	0.01-2V	560 mA g^{-1}	100 cycles, 190 mA h g^{-1}	[S6]
S, O-doped porous hard carbon microspheres	0.01-2.5V	1000 mA g^{-1}	2000 cycles, 108.4 mA h g-1	[S7]
amorphous ordered mesoporous carbon	0.01-2.5V	1000 mA g^{-1}	1000 cycles, 146.5 mA h g^{-1}	[S8]
core-shell structured N, O-doped hollow porous carbon microspheres	0.01-2.5V	5000 mA g^{-1}	6000 cycles, 202.6 mA h g^{-1}	This work

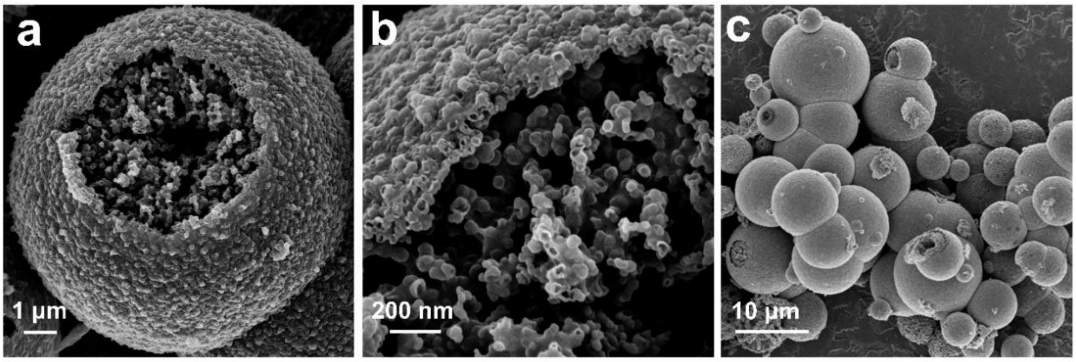

Fig. S1 SEM image of NOHPC

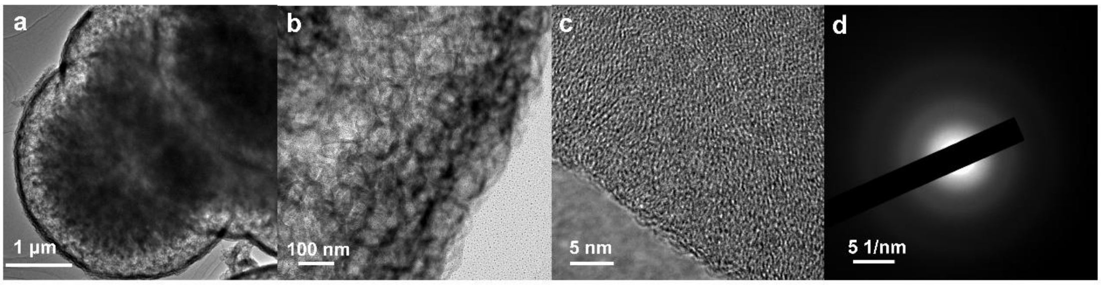

Fig. S2 (**a, b**) TEM images of NOHPC. (**c**) HRTEM image of NOHPC. (**d**) SAED pattern of NOHPC

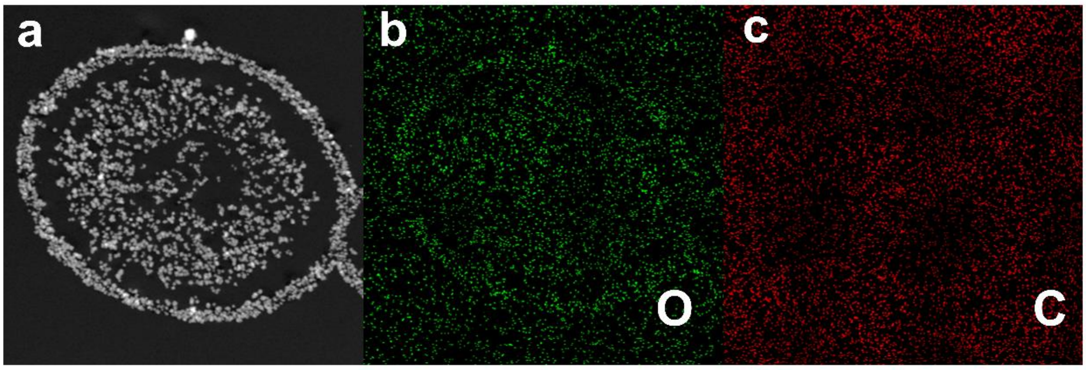

Fig. S3 EDS elemental mapping images of ultrathin section for unwashed NOHPC

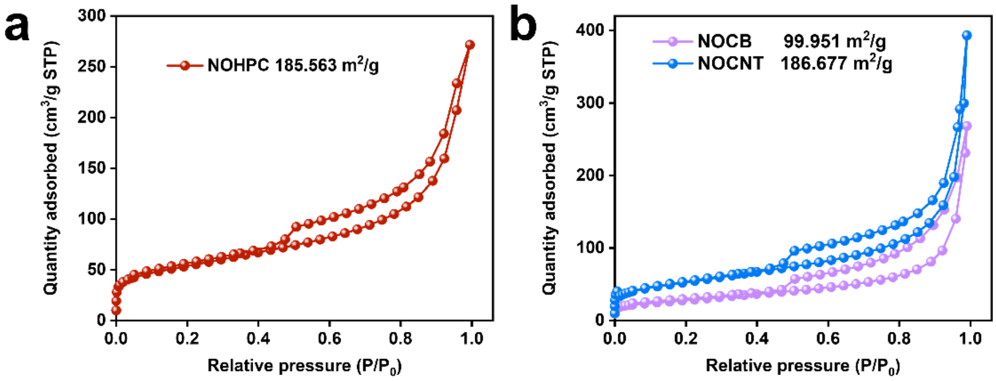

Fig. S4 (**a**) Nitrogen adsorption–desorption isotherms of NOHPC; (**b**) Nitrogen adsorption–desorption isotherms of NOCB and NOCNT

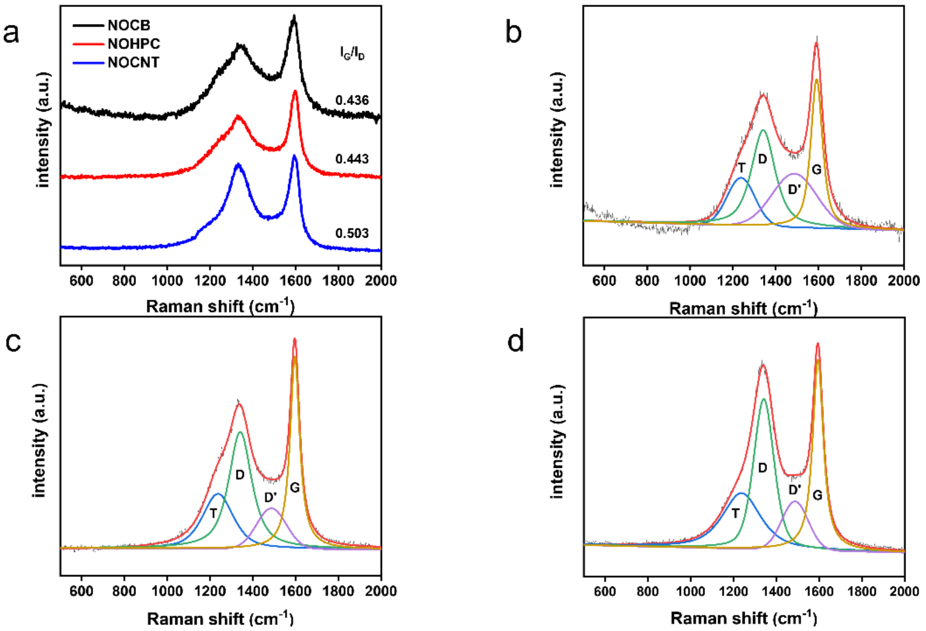

Fig. S5 (**a**) Raman spectra curves of NOCB, NOHPC and NOCNT. Fitted Raman spectra curves of (**b**) NOCB, (**c**) NOHPC, (**d**) NOCNT. The green line with a peak at 1360 cm^{-1} represents the D-band, the purple line with a peak at 1500 cm^{-1} represents the D'-band, while the yellow line with a peak close to 1585 cm^{-1} represents the G-band. Integrated ratios are obtained from the area of the fitted peaks (ratio of the area of G-band to the sum of the area of D-band and D'-band). The fitting is made by using the OMINC Picta software program with a guassian-laurenztian fit [S9]

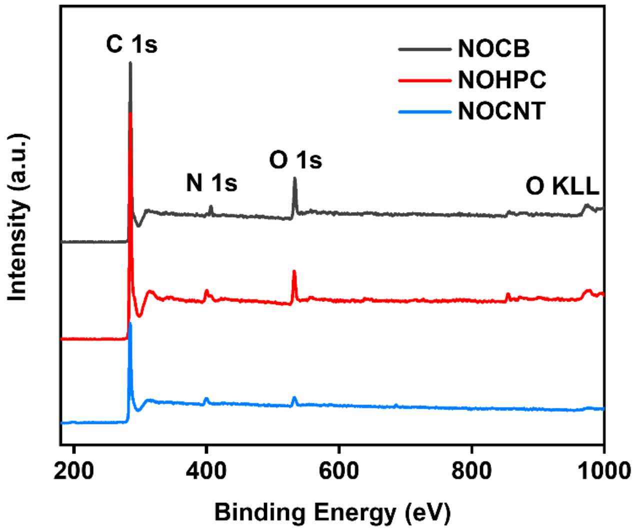

Fig. S6 XPS survey of NOCB, NOHPC and NOCNT

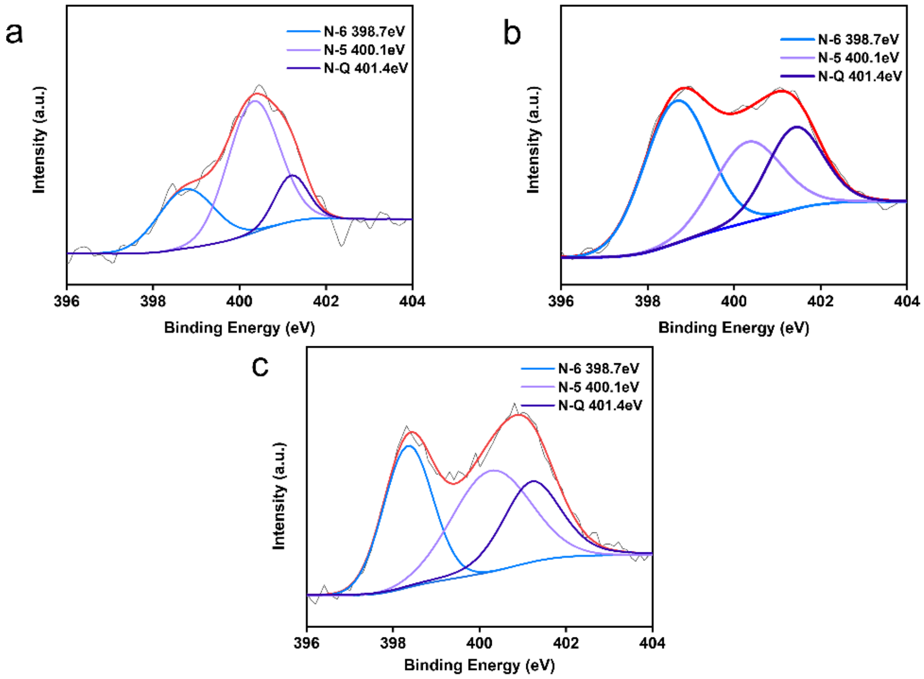

Fig. S7 High-resolution N 1s spectra of (**a**) NOCB, (**b**) NOHPC and (**c**) NOCNT

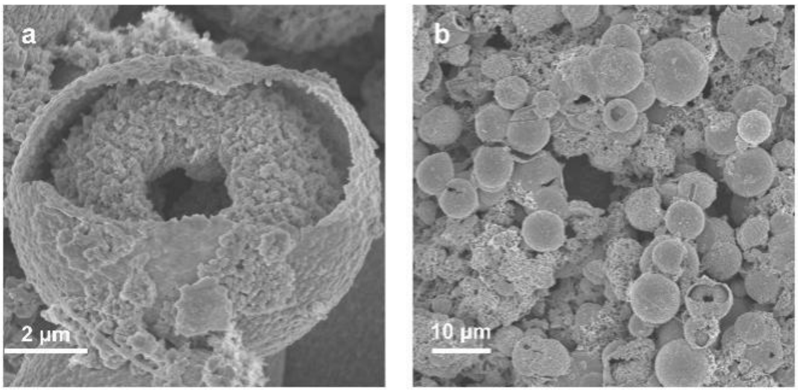

Fig. S8 SEM images of NOHPC anode after 500 cycles at 0.5 A g^{-1}

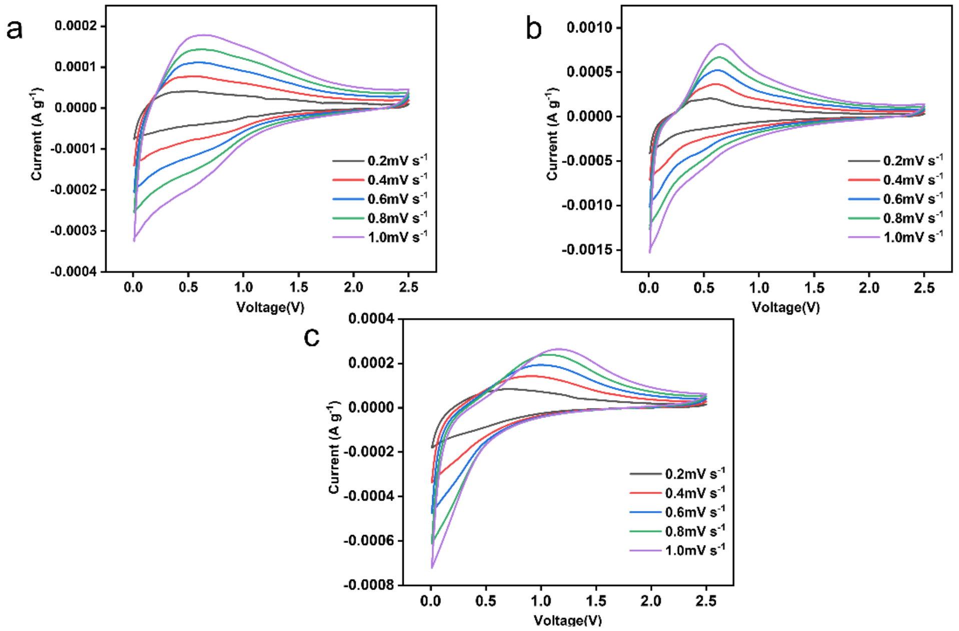

Fig. S9 CV curves of (**a**) NOCB, (**b**) NOHPC, (**c**) NOCNT at different scanning rates range from 0.2 to 1.0 mV s^{-1}

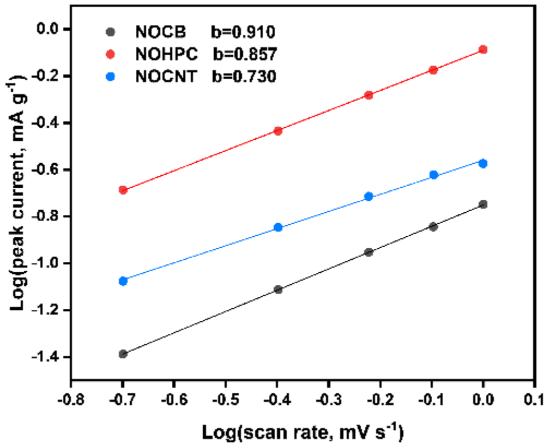

Fig. S10 The logarithm relationship between the scan rates and the anodic peak current

The current responses of one certain electrode process are from two kinds of contribution, capacitive and diffusion-limited processes. The fast capacitive contributions originate from the surface charge-transfer, chemical adsorption and Faradaic process occur in a thin layer of electrode materials. The current response from a cyclic voltammogram obeys the relationship of equation.

$$i = av^b$$

where *a* and *b* are adjustable parameters. For the ideal capacitive behavior, the current response is proportional to the scan rate (b = 1). The current response of diffusionlimited process is proportional to the square root of the scan rate ($b = 0.5$). The adjustable parameters of *a* and *b* can be calculated using the linear of *lgi* and *lgv* with the following equation.

$$lgi = lga + blgv$$

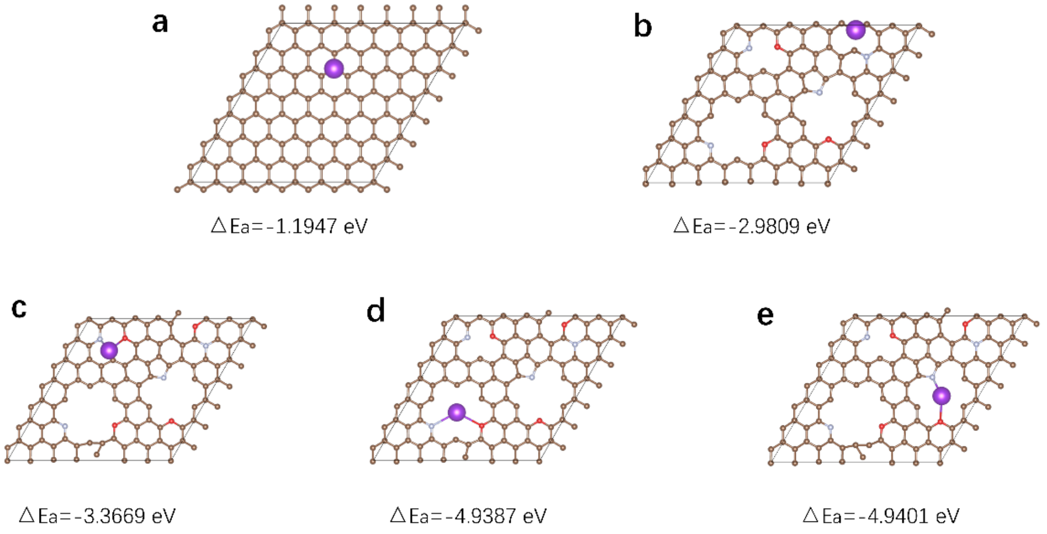

Fig. S11 The optimized model and top views of K atom absorbed in the (**a**) pristine, (**b**) S1: N-Q/O-doped, (**c**) S2: N-6/O-doped, (**d**) S3: N-6/O-doped and (**e**) S4: N-5/O-doped carbon structures and their corresponding ΔEa

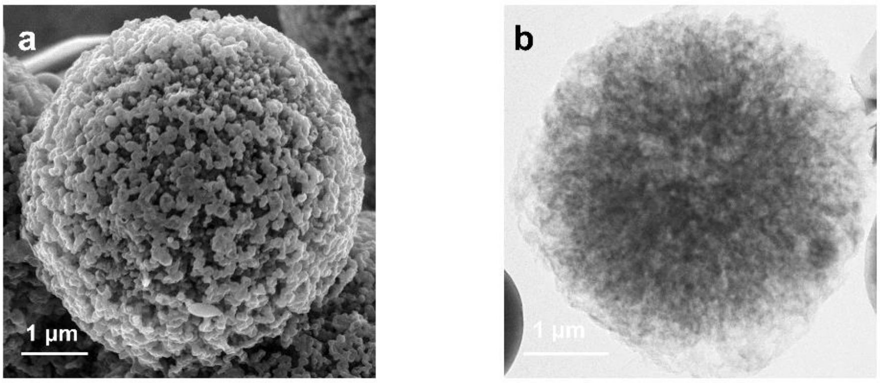

Fig. S12 (**a**) SEM image of HPAC. (**b**) TEM images of HPAC

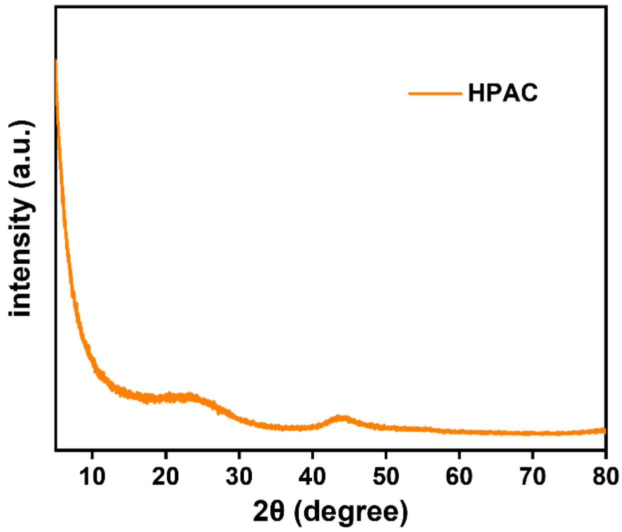

Fig. S13 XRD patterns of HPAC

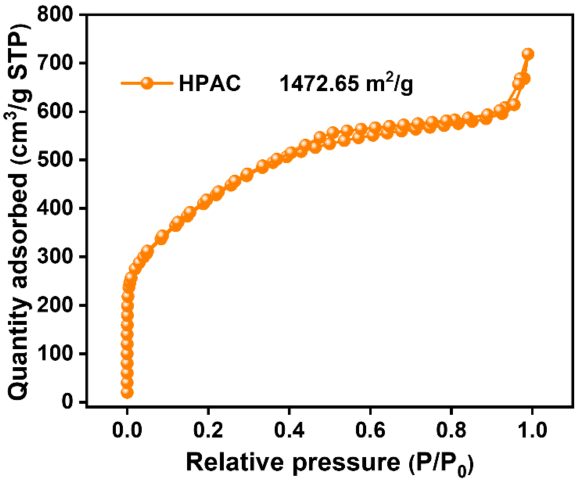

Fig. S14 Nitrogen adsorption-desorption isotherms of HPAC

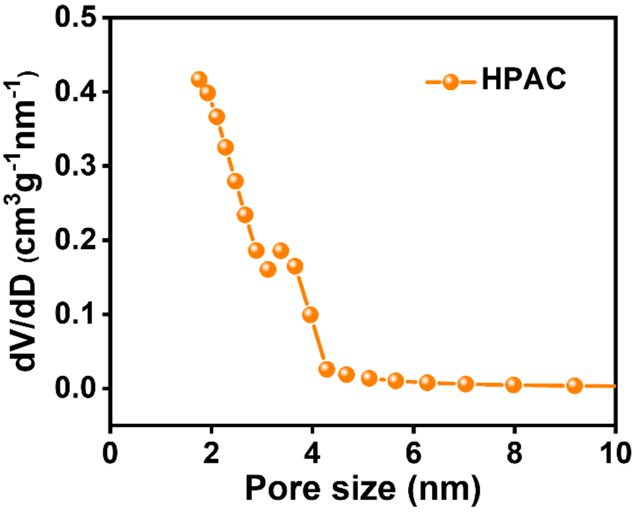

Fig. S15 BJH pore width of HPAC

Supplementary References

[S1] G. Kresse, D. Joubert, From ultrasoft pseudopotentials to the projector augmented-wave method. Phys. Rev. B **59**, 1758 (1999). https://doi.org/10.1103/PhysRevB.59.1758

[S2] J.P. Perdew, K. Burke, M. Ernzerhof, Generalized gradient approximation made simple. Phys. Rev. Lett. **77**, 3865 (1996). https://doi.org/10.1103/PhysRevLett.77.3865

[S3] S. Grimme, J. Antony, S. Ehrlich, H. Krieg, A consistent and accurate ab initio parametrization of density functional dispersion correction (DFT-D) for the 94 elements H-Pu. J. Chem. Phys. **132**, 154104 (2010). https://doi.org/10.1063/1.3382344

[S4] X.-S. Tao, Y.-G. Sun, Y. Liu, B.-B. Chang, C.-T. Liu et al., Facile synthesis of hollow carbon nanospheres and their potential as stable anode materials in potassium-ion batteries. ACS Appl. Mater. Interfaces **12**, 13182-13188 (2020). https://doi.org/10.1021/acsami.9b22736

[S5] D. Qiu, J. Guan, M. Li, C. Kang, J. Wei et al., Kinetics enhanced nitrogen-doped hierarchical porous hollow carbon spheres boosting advanced potassium-ion hybrid capacitors. Adv. Funct. Mater. **29**, 1903496 (2019). https://doi.org/10.1002/adfm.201903496

[S6] Z. Jian, Z. Xing, C. Bommier, Z. Li, X. Ji, Hard carbon microspheres: potassium-ion anode versus sodium-ion anode. Adv. Energy Mater. **6**, 1501874 (2016). https://doi.org/10.1002/aenm.201501874

[S7] M. Chen, W. Wang, X. Liang, S. Gong, J. Liu et al., Sulfur/oxygen codoped porous hard carbon microspheres for high-performance potassium-ion batteries. Adv. Energy Mater. **8**, 1800171 (2018). https://doi.org/10.1002/aenm.201800171

[S8] W. Wang, J. Zhou, Z. Wang, L. Zhao, P. Li et al., Short-range order in mesoporous carbon boosts potassium-ion battery performance. Adv. Energy Mater. **8**, 1701648 (2018). https://doi.org/10.1002/aenm.201701648

[S9] A. Sadezky, H. Muckenhuber, H. Grothe, R. Niessner, U. Pöschl, Raman microspectroscopy of soot and related carbonaceous materials: Spectral analysis and structural information. Carbon **43**, 1731-1742 (2005). https://doi.org/10.1016/j.carbon.2005.02.018
